# Evaluating the induced photon contamination by different breast IOERT shields using Monte Carlo simulation

**DOI:** 10.1002/acm2.14098

**Published:** 2023-07-18

**Authors:** Hamid Reza Baghani, Mostafa Robatjazi

**Affiliations:** ^1^ Physics Department Hakim Sabzevari University Sabzevar Iran; ^2^ Medical Physics and Radiological Sciences Department Sabzevar University of Medical Sciences Sabzevar Iran; ^3^ Non‐communicable Disease Research Center Sabzevar University of Medical Sciences Sabzevar Iran

**Keywords:** breast cancer, electron beam, intraoperative radiotherapy, Monte Carlo simulation, photon contamination, radioprotection disk

## Abstract

**Background and Objective:**

Avoiding the underlying healthy tissue over‐exposure during breast intraoperative electron radiotherapy (IOERT) is owing to the use of some dedicated radioprotection disks during patient irradiation. The originated contaminant photons from some widely used double‐layered shielding disks including PMMA+Cu, PTFE+steel, and Al+Pb configurations during the breast IOERT have been evaluated through a Monte Carlo (MC) simulation approach.

**Methods:**

Produced electron beam with energies of 6, 8, 10, and 12 MeV by a validated MC model of Liac12 dedicated IOERT accelerator was used for disk irradiations. Each of above‐mentioned radioprotection disks was simulated inside a water phantom, so that the upper disk surface was positioned at R_90_ depth of each considered electron energy. Simulations were performed by MCNPX (version 2.6.0) MC code. Then, the energy spectra of the contaminant photons at different disk surfaces (upper, middle, and lower one) and relevant contaminant dose beneath the studied disks were determined and compared.

**Results:**

None of studied shielding disks show significant photon contamination up to 10 MeV electron energy, so that the induced photon dose by the contaminant X‐rays was lower than those observed in the disk absence under the same conditions. In return, the induced photon dose at a close distance to the lower disk surface exceeded from calculated values in the disk absence at 12 MeV electron energy. The best performance in contaminant dose reduction at the energy range of 6–10 MeV belonged to the Al+Pb disk, while the PMMA+Cu configuration showed the best performance in this regard at 12 MeV energy.

**Conclusion:**

Finally, it can be concluded that all studied shielding disks not only don't produce considerable photon contamination but also absorb the originated X‐rays from electron interactions with water at the electron energy range of 6–10 MeV. The only concern is related to 12 MeV energy where the induced photon dose exceeds the dose values in the disk absence. Nevertheless, the administered dose by contaminant photons to underlying healthy tissues remains beneath the tolerance dose level by these organs at the entire range of studied electron energies.

## 1 INTRODUCTION

Breast cancer radiotherapy using high‐energy electrons originating from dedicated intraoperative accelerators has gained a special interest in the last two decades.^1^, ^2^, ^3^, ^4^ In this method, the patient has undergone irradiation immediately after the surgery and when the patient is under anesthesia.^5^, ^6^ To do so, at first, the gross tumor volume and its positive surrounding margins are removed through the surgery and the remaining tumor bed is irradiated by high‐energy electrons (up to 12 MeV) which are produced by currently introduced portable and dedicated linacs such as Liac, Novac, and Mobetron for intraoperative radiotherapy (IORT) purposes.^7^, ^8^, ^9^ The main features of these dedicated IORT accelerators are very high dose rate capability and very low photon contamination which respectively minimize the treatment time and provide employing these machines in a standard and unshielded operating theatre for patient irradiation.^10^, ^11^, ^12^ Since the electron beam is directly used for patient irradiation, this method is known as intraoperative electron radiotherapy (IOERT).^13^

According to the fact that the underlying healthy tissues such as ribs, superficial pectoral muscles, lung, and heart (in the case of left‐sided breast IOERT) may be directly exposed to the incident electron beam during the breast intraoperative electron radiotherapy, one should shield these organs at risk (OARs) during the radiotherapy in order to minimize the risk of normal tissue complications after the radiotherapy.^14^, ^15^, ^16^, ^17^ Accordingly, a dedicated radioprotection disk must be used during the breast IOERT to avoid overexposure of the underlying healthy tissues. Due to the intervention of this radioprotection disk during the breast IOERT, the radiotherapy procedure is performed as follows; the remaining tumor bed after the surgery is prepared as a cylindrical flap which is directly located upon the radioprotection disk. Then, the depth of the prepared flap (which is considered as the planning target volume (PTV) during the breast IOERT) is measured by the surgeon and the intraoperative electron energy is selected according to the flap depth in such a way that the 90% isodose level fully cover the distal end of the tumor bed.^18^ Afterward, a proper cylindrical applicator is used as the beam collimation system through which the intraoperative electron beam is delivered to the prepared flap by the surgeon (the PTV, in other words).^2^, ^19^ Totally, the size of the applicator is about two centimeters greater than the flap diameter and the disk diameter is also one centimeter larger than the employed applicator diameter.

The employed radioprotection disks during the breast IOERT are mainly designed and constructed in a double‐layered format.^15^ The first layer (which is exposed to the incident electron beam) is made from a low atomic number material (such as PMMA, Teflon, or Aluminum (Al)) to moderate and stop the transmitted electrons from the PTV without producing a remarkable photon contamination through bremsstrahlung process. On the other hand, the second layer (which is faced to the underlying healthy tissues) is generally constructed from a high atomic number material (such as Lead (Pb), stainless steel, or Copper (Cu)) to attenuate and absorb the produced bremsstrahlung photons by the first layer (low atomic number one).^14^, ^20^

Up to now, different double‐layered disk compositions including PMMA+Cu, Al+Pb, and PTFE (Polytetrafluoroethylene, a synthetic type of Teflon)+steel have been used as the radioprotection disk during the breast cancer IOERT.^15^

In our previous study, the electron‐based dosimetric characteristics of the above‐mentioned radioprotection disks including backscatter factor (BSF), transmission factor (TF), and variations of central depth dose curves in the presence and absence of each studied disk were fully evaluated and compared.^15^ Nevertheless, one of the main concerns relevant to these dedicated radioprotection disks is the induced photon contamination during the radiotherapy which can lead to the considerable biologic effects and secondary complications for underlying healthy tissues following the intraoperative radiotherapy. Although the double‐layered structure of the employed radioprotection disk can considerably reduce these radiation contamination components, they may be still produced when the transmitted electron beam from the target volume encounters with the radioprotection disk. In this regard, the current study aims to evaluate and compare the induced photon contamination by different introduced radioprotection disks for intraoperative electron radiotherapy (PMMA+Cu, PTFE+steel, Al+Pb). To accomplish this, a Monte Carlo (MC) simulation approach was considered and self‐induced radiation contamination at different surfaces of each shielding disk (upper, middle, and lower) was scored and compared for various nominal intraoperative electron energies. Finally, the variations of contaminant photon dose component beneath each considered IOERT shielding disk were determined and compared.

## 2 MATERIALS AND METHODS

### 2.1 Dedicated IOERT accelerator

Liac 12 MeV model, a dedicated IOERT accelerator, was employed in the current study. This accelerator is introduced by Sordina (Italy) company and designed in such a way that it can produce the electron beam with nominal energies of 6, 8, 10, and 12 MeV.^21^ This accelerator is equipped with some PMMA‐made cylindrical applicators for electron beam collimation and radiation dose delivery to the patient which can connect the irradiated volume to the accelerator head through the hard docking mechanism.^12^ The length of all employed applicators is equal to 60 cm. The diameter of these cylindrical applicators can change from 3 to 10 cm, while their base angle varies between 0 and 45°). The flat‐based applicator (zero degrees bevel angle) with a 10 cm diameter is introduced as the reference applicator.^22^, ^23^ This accelerator can produce high dose rates up to 30 Gy/min by adjusting the machine PRF (pulse repetition frequency) between 1 and 60 Hz. The provided SSD (source to surface distance) by this accelerator is 71.3 cm which is measured from an Aluminum‐made scattering foil to the distal end of the employed applicator.^24^

MCNPX (version 2.6) was used in the current study for simulating the intraoperative electron irradiations and relevant photon contamination beneath the studied shielding disks.^25^, ^26^ In this regard, A Validated Monte Carlo‐simulated Model of Liac12 head at different intraoperative electron energies was employed.^27^ The simulated modules respectively included a Titanium (Ti) exit window, Al scattering foil, air‐filled monitor ion chambers, a Mylar‐made protective layer, sidelong PEEK and PMMA polymer support, and a cylindrical PMMA‐based applicator. The initial electron energy spectra and their spatial distribution upon the exit window were provided by the manufacturer (Sordina, SpA, Italy). The validity of the employed Monte Carlo model of the Liac12 head at different nominal electron energies has been confirmed through comparing the relevant PDD (percentage depth dose) and TDP (transverse dose profiles) data with those practically measured through ionometric dosimetry inside a water phantom.^27^

### 2.2 Employed IOERT shielding disks

As mentioned previously, three different types of double‐layered IOERT radioprotection disks were considered in the current study. The main features of these radioprotection disks including the layer thickness, composing materials, and their relevant physical densities have been reported in Table 1.^15^

**TABLE 1:** The fundamental features of employed radioprotection disks at the current study.

	Feature			
Shield type	Thickness	Density (g/cm^3^)	First layer (face to radiation)	Second layer (face to distal end of tumor bed)
PTFE+ steel	3 mm PTFE+ 3 mm steel	2.25 (PTFE); 8.06 (steel)	PTFE	Steel
PMMA+ Cu	7 mm PMMA+ 3 mm Cu	1.19 (PMMA); 8.96 (Cu)	PMMA	Cu
Al+ Pb	4 mm Al+ 2 mm Pb	2.70 (Al); 11.35 (Pb)	Al	Pb

These radioprotection disks are the most widespread ones which are clinically used during breast intraoperative electron radiotherapy to minimize both the received dose by the healthy underlying tissues and the electron backscattered dose originating from the upper disk surface.^20^, ^28^ As demonstrated in Table 1, the thickness of these disks does not exceed 1 cm, because higher disk thicknesses may not allow the surgeon to prepare an appropriate flap (remaining the tumor bed after the surgery) upon the disk surface.

Besides, as provided in Table 1, the first disk layer is always constructed from a low atomic number element or composition with a light physical density to ensure minimizing the electron backscattering effect from the upper disk surface. This backscatter dose component can considerably affect the dose distribution uniformity inside the target volume, as established by some studies.^14^, ^15^, ^16^, ^20^, ^28^, ^29^, ^30^

### 2.3 Evaluating the photon contamination

**FIGURE 1:**
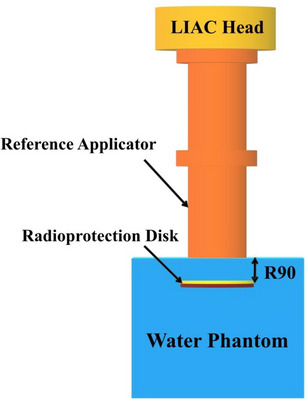
Schematic diagram of the simulated LIAC12 head along with 10 cm reference applicator and inserted radioprotection disk inside the water phantom at the depth of R_90_.

To evaluate the induced photon contamination by each considered radioprotection disk at different intraoperative electron energies of 6, 8, 10, and 12 MeV, the disk was located at the R_90_ depth (the depth at which the electron dose reaches 90% of the maximum absorbed dose) along the beam central axis related to each studied electron energy inside the water. The reason for considering this depth was that (1) the electron energy in IOERT is selected in such a way that the 90% isodose line fully covers the distal end of the tumor bed and (2) the electron dose in breast cancer IOERT is generally prescribed at the depth of R_90_.^18^ Accordingly, this depth was considered to be as close as possible to the conditions which exist in clinical practice. All of the disk irradiations were performed inside a water phantom with dimensions of 30 × 30 × 8 cm^3^ and employing the reference applicator (flat‐based cylindrical applicator with 10 cm diameter). The schematic diagram of the employed irradiation condition in presence of each studied radioprotection disk has been illustrated in Figure 1.

To evaluate the induced photon contamination by each radioprotection disk at different intraoperative electron energies of 6, 8, 10, and 12 MeV, the relevant energy spectrum of contaminant photons was scored at different disk surfaces (upper surface, middle one, and lower surface). To accomplish this, the F2 scoring tally, a dedicated tally for scoring the particle fluence over a surface in MCNPX Monte Carlo code, was used.^25^ Then, the mean contaminant photon energy and the total number of contaminant photons at each disk surface (per one incident electron) were determined for each considered IOERT shield.

It has been previously shown that the water phantom itself can produce some photon contamination at different nominal intraoperative electron energies.^31^ Therefore, to accurately quantify the induced radiation contamination by the disk itself, two simulation schemes including the presence and absence of the IOERT shield were taken in the current research, and scored radiation contaminations for each disk layer were directly compared with those calculated at the corresponding depth in the absence of radioprotection disk.

In addition to the contaminant energy spectra, the induced absorbed dose by contaminant photons beneath each evaluated disk was also determined at different electron energies. To do so, the deposited photon energy was scored inside some water‐filled cubic dosimetry cells with the dimensions of 5 × 5 × 2 mm^3^ which were consecutively located along the electron beam central axis. It should be mentioned that the *F8 scoring tally (energy deposition within a defined cell), the standard tally for radiation dosimetry in MCNPX Monte Carlo code, was used for this purpose.^25^ Finally, the calculated energy deposition inside each scoring cell was divided by its mass to obtain the absorbed dose in terms of Gray (Gy). Then, the absorbed dose values were converted to the equivalent dose (in terms of Sievert (Sv)) by considering the relevant radiation weighting factor (w_R_) which is equal to unity for photon beam at all energies.^32^

In all of the modeled geometry media including Laic12 head and connected applicator, water phantom, and radioprotection disk, both electrons and photons were fully transported. The energy cut‐off during the electron and photon transport was correspondingly set to 50 and 10 keV. Both photon and electron interaction libraries were taken from the ENDF/B‐VI data file. The electron and photon cross‐sections were respectively taken from mcplib04 and el03 data tables. The Monte Carlo simulations were performed by a CPU Intel® Corei7/8 GB RAM computing system and 10^9^ particle histories were followed in each performed simulation to reduce the associated statistical uncertainty with the obtained MC results to less than 0.5%.

## 3 RESULTS AND DISCUSSION

**FIGURE 2:**
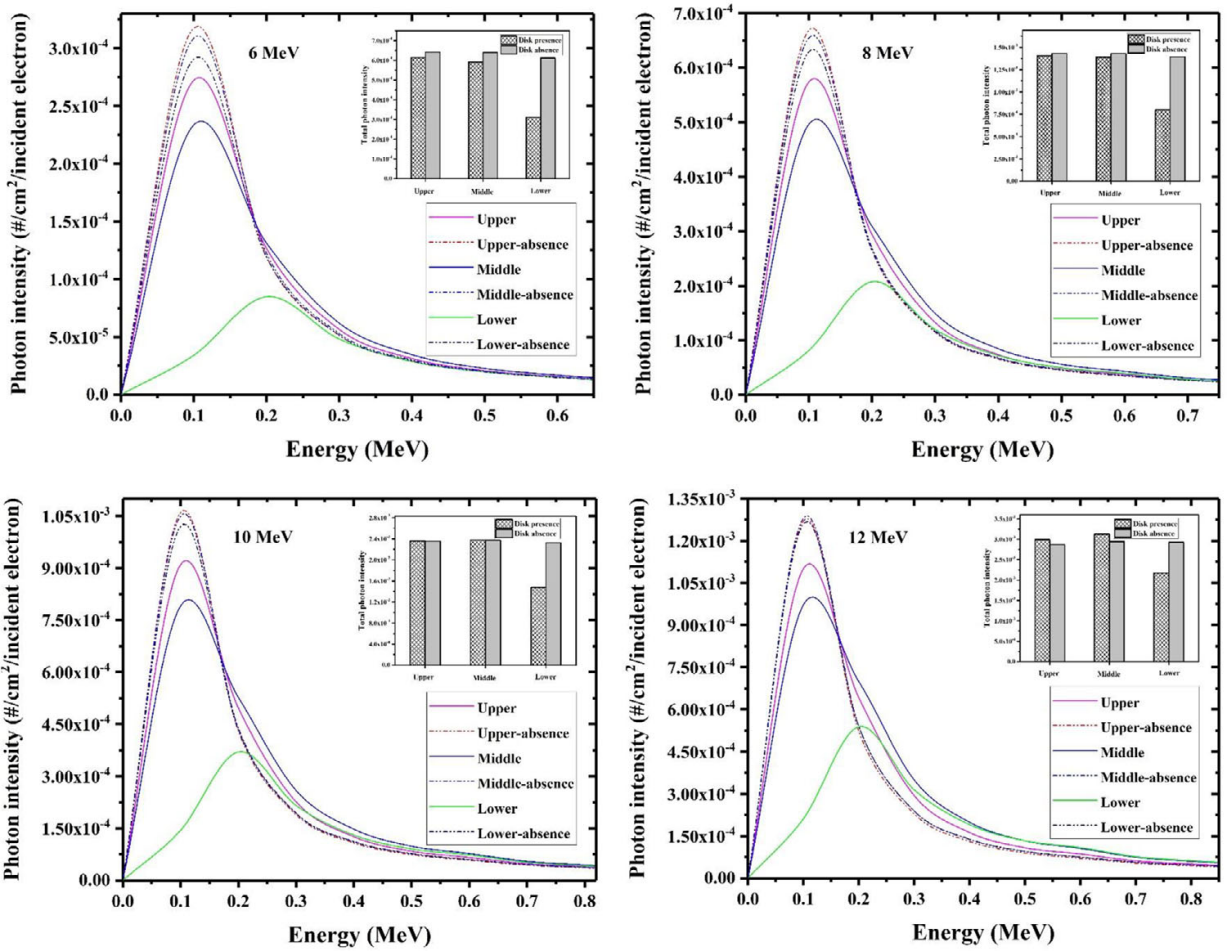
The energy spectra relevant to the contaminant photons in absence and presence of the PMMA+ Cu radioprotection disk at various nominal electron energies which have been scored at different locations including the top disk surface, middle surface and lower one. The total number of scored photons at each disk surface has been also appended the illustrated spectra at different energies (as shown with bar charts).

**FIGURE 3:**
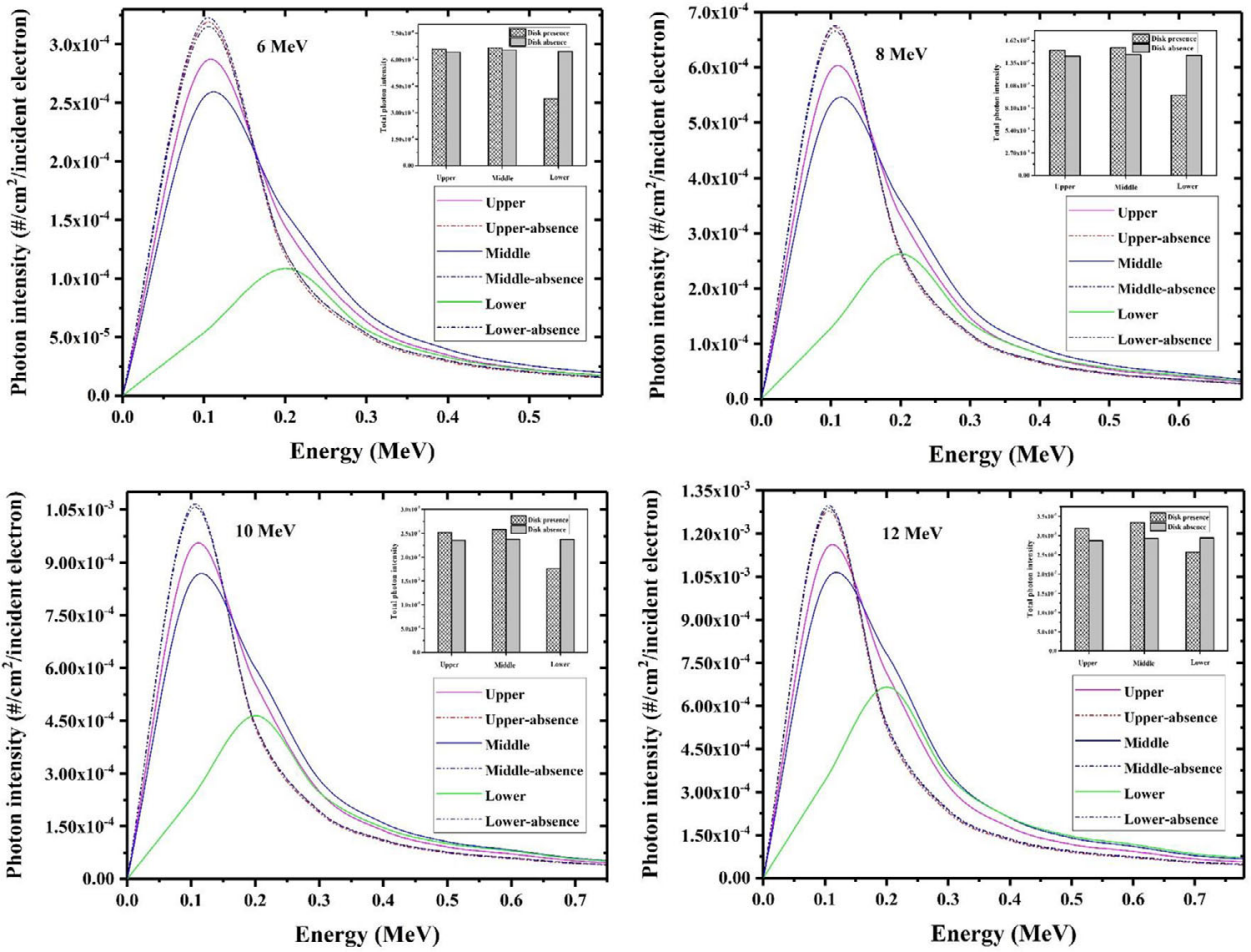
The energy spectra relevant to the contaminant photons in absence and presence of the PTFE+ Steel radioprotection disk at various nominal electron energies which have been scored at different locations including the top disk surface, middle surface and lower one. The total number of scored photons at each disk surface has been also appended the illustrated spectra at different energies (as shown with bar charts).

**FIGURE 4:**
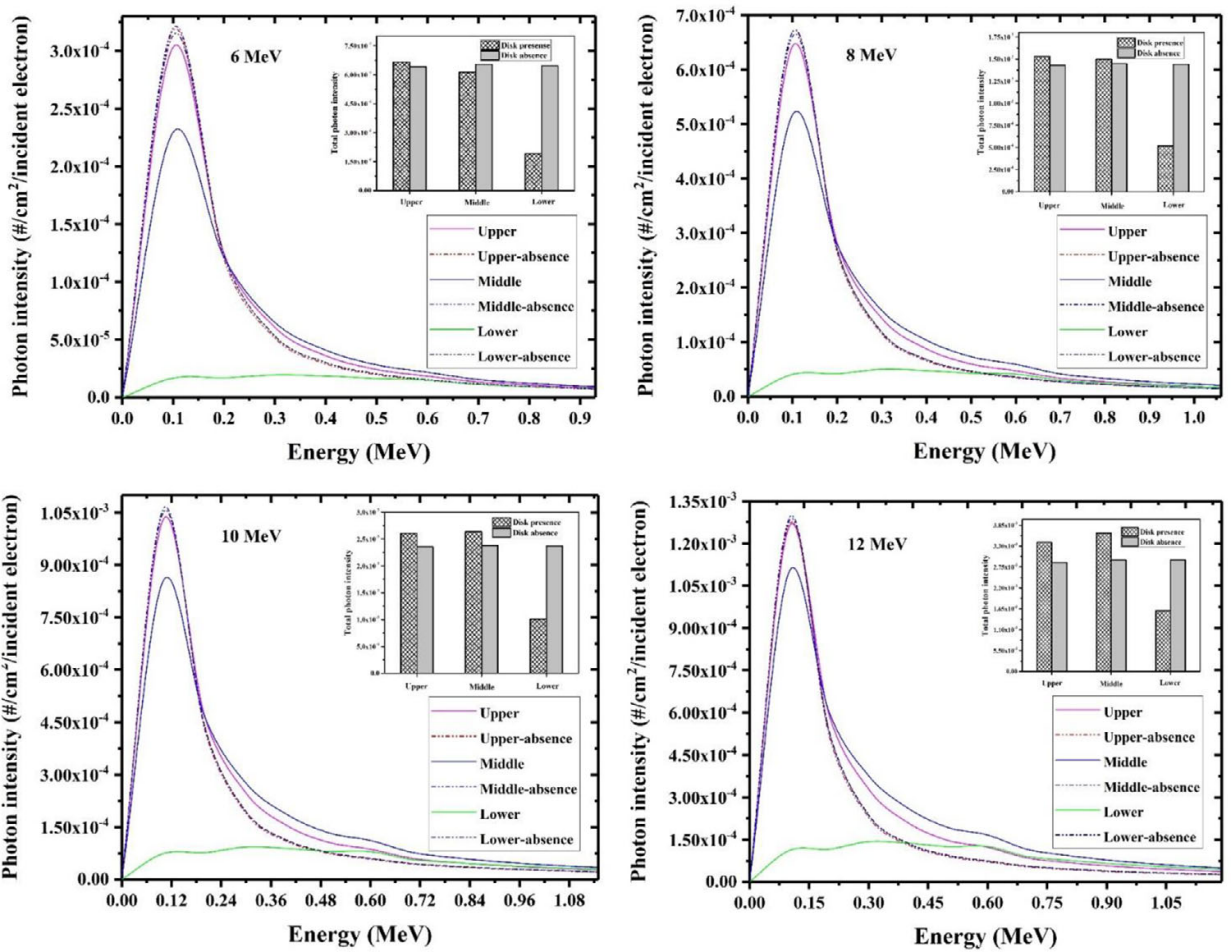
The energy spectra relevant to the contaminant photons in absence and presence of the Al+ Pb radioprotection disk at various nominal electron energies which have been scored at different locations including the top disk surface, middle surface and lower one. The total number of scored photons at each disk surface has been also appended the illustrated spectra at different energies (as shown with bar charts).

The calculated contaminant photon energy spectra at different surfaces of each studied radioprotection disk including PMMA+Cu, PTFE+steel, and Al+Pb have been respectively shown in Figures 2, 3, 4 (solid lines). As illustrated, the photon contamination spectra in the absence of each considered shielding disk have been also depicted (dash‐dot lines) to better realize the magnitude of the photon contamination which is induced by the radioprotection disk itself. Furthermore, the total number of contaminant photons (per one incident electron) in the presence and absence of each disk at different surfaces have been also appended to these Figures, as shown with bar charts.

It should be mentioned that the reported photon energy spectra in the absence of the radioprotection disk are relevant to those which are directly produced through electron interactions inside the water phantom.^31^

As illustrated in Figure 2, the amount of photon contamination at all surfaces of the PMMA+Cu radioprotection disk remains lower than those that exist in the absence of the disk, up to 8 MeV intraoperative electron energy. This means that the presence of this disk not only does not increase the rate of photon contamination but also absorbs the contaminant photons originating from the electron interactions with the water. This finding is due to the low energy of the incident electrons as well as their considerable energy loss before impinging on the upper disk surface which can finally reduce the probability of the bremsstrahlung photon production to a negligible amount. In addition, the produced low contaminant X‐rays will be again absorbed by the high atomic number layer of the radioprotection disk (the lower disk layer). At 10 and 12 MeV electron energy, the intensity of contaminant photons at the upper and middle surfaces of the PMMA+Cu disk is near to those that exist in the absence of this IOERT shield and may also exceed calculated values in the absence of the disk (especially at 12 MeV electron energy). Nevertheless, the intensity of the photon contamination at the lower disk surface is always lower than the scored values at the disk absence. This issue also realizes that a remarkable portion of produced X‐rays inside the disk body will be reabsorbed by the lower layer of the radioprotection disk.

As depicted in Figure 2, the maximum intensity of the photon contamination at both upper and middle surfaces of the PMMA+Cu disk is seen at about 0.1 MeV (regardless of the incident electron energy), while this value shifts to about 0.2 MeV energy for the lower surface of PMMA+Cu radioprotection disk. This fact can be mainly linked to the increased severity of the beam hardening effect by the disk itself when moving toward the lower surfaces.

As shown in Figure 3, the intensity of the contaminant photons at the upper and middle surfaces of the PTFE+steel radioprotection disk is always higher than the calculated ones in the disk absence for all studied electron energies. This finding means that the presence of this IOERT shield can amplify the intensity of the contaminant photons through electron interactions with the disk body. Nonetheless, as demonstrated in Figure 3, the final intensity of the photon contamination at the lower disk surface is lower than that of disk absence at all energies. This result indicates the effective role of the steel layer in reabsorbing a significant part of the produced contaminant X‐rays.

As depicted in Figure 3, the maximum contaminant photon intensity at upper and middle surfaces of the PTFE+steel radioprotection disk is observed at about 0.1 MeV energy, while this value increments to about 0.2 MeV at the lower disk surface for all studied intraoperative electron energies. This energy increase can be also attributed to the beam hardening effect as discussed earlier.

An analogous trend, as seen for the PTFE+steel disk, was also found for the Al+Pb IOERT shield (as shown in Figure 4), so that the intensity of the photon contamination at both upper and middle surfaces of this disk was higher than the corresponding scored values at the disk absence for all electron energies. On the contrary, the intensity of the contaminant photons at the lower surface of the Al+Pb disk was always lower than the calculated one in the disk absence. This finding also demonstrates the desirable performance of the Lead layer in the absorption of the contaminant X‐rays.

The maximum difference between the scored photon contamination intensity at the lower surface of the disk and the corresponding calculated value at the disk absence is observed for the Al+Pb shield, such that the presence of this disk will reduce the photon intensity by about 70.6%, 63.9%, 57.4%, and 45.9% at 6, 8, 10, and 12 MeV electron energy, respectively. On the other hand, this photon intensity reduction for PMMA+Cu and PTFE+steel radioprotection disks respectively lies within 49.1%−25.7% and 41.3%−12.6% with increasing the intraoperative electron energy.

As illustrated in Figure 4, the energy distribution of the contaminant photons at both upper and middle surfaces of the Al+Pb disk is also centralized at about 0.1 MeV for all incident electron energies. In return, unlike two previous IOERT shields, the energy distribution of the contaminant photons at the lower disk surface is approximately uniform and shows a similar photon intensity at different energies. This issue is mainly due to the remarkable attenuation of the contaminant X‐rays by the lower Pb layer which can practically lead to fading the observed peak in contaminant photon energy spectra. Besides, this heavy X‐ray attenuation has an impressive effect in eliminating the very low energy part of the scored X‐ray spectra at the prior disk layers (upper and middle ones).

For all radioprotection disks understudy, the photon contamination intensity at all disk surfaces increments with an increase in the intraoperative electron energy. For example, the intensity of the contaminant X‐rays at the middle surface of the PMMA+Cu, PTFE+steel, and Al+Pb correspondingly increments by a factor of about 4.0, 4.3, and 4.9 when electron energy increases from 6 MeV to 12 MeV. This result can be justified by the increased probability of the bremsstrahlung process at higher electron energies.

From the reported photon contamination data in Figures 2–4, it can be deduced that the intensity of the photon contamination at both upper and middle surfaces of the Al+Pb disk is higher than those of two other studied radioprotection disks, while the lowest photon contamination values for these two surfaces (upper and middle ones) are seen for PMMA+Cu shield. This result means that the maximum values of photon contamination originate from the Al+Pb disk which is a reasonable finding viewpoint to the higher atomic number and density of the contributing elements in the construction of this radioprotection disk (as reported in Table 1). On the other hand, the minimum and maximum intensity of the photon contamination at the lower disk surface would be found for Al+Pb and PTFE+steel, respectively. Although the Al+Pb disk shows the most significant role in photon contamination, this finding indicates that the Al+Pb disk also has the most contribution to the reabsorption of these contaminant X‐rays because this radioprotection disk reduces the photon contamination intensity to the minimum level among the studied radioprotection disks at all intraoperative electron energies.

The mean energy of the contaminant X‐rays at different surfaces of studied radioprotection disks including PMMA+Cu, PTFE+steel, and Al+Pb along with the corresponding values in the absence of each shielding disk has been listed in Tables 2, 3, 4, respectively.

**TABLE 2:** The mean contaminant photon energy acquired at different layers of PMMA+Cu radioprotection disk at 6, 8, 10, and 12 MeV intraoperative electron energy.

	Mean contaminant photon energy (MeV)					
	Disk presence			Disk absence		
Electron energy (MeV)	Upper surface	Middle surface	Lower surface	Upper surface	Middle surface	Lower surface
6	0.403	0.418	0.624	0.384	0.384	0.389
8	0.488	0.503	0.725	0.469	0.468	0.471
10	0.557	0.572	0.797	0.539	0.536	0.539
12	0.602	0.617	0.824	0.591	0.587	0.588

**TABLE 3:** The mean contaminant photon energy acquired at different layers of PTFE+steel radioprotection disk at 6, 8, 10, and 12 MeV intraoperative electron energy.

	Mean contaminant photon energy (MeV)					
	Disk presence			Disk absence		
Electron energy (MeV)	Upper surface	Middle surface	Lower surface	Upper surface	Middle surface	Lower surface
6	0.395	0.408	0.572	0.384	0.382	0.383
8	0.477	0.491	0.666	0.469	0.467	0.467
10	0.544	0.558	0.734	0.539	0.538	0.537
12	0.588	0.605	0.762	0.591	0.587	0.588

**TABLE 4:** The mean contaminant photon energy acquired at different layers of Al+Pb radioprotection disk at 6, 8, 10, and 12 MeV intraoperative electron energy.

	Mean contaminant photon energy (MeV)					
	Disk presence			Disk absence		
Electron energy (MeV)	Upper surface	Middle surface	Lower surface	Upper surface	Middle surface	Lower surface
6	0.396	0.440	0.905	0.384	0.382	0.383
8	0.481	0.528	1.044	0.469	0.466	0.467
10	0.551	0.598	1.138	0.539	0.537	0.537
12	0.605	0.658	1.173	0.591	0.587	0.588

As indicated in Tables 2, 3, 4, the mean contaminant photon energy increments with moving toward the lower surface of each considered radioprotection disk at the same incident electron energy (i.e. this value respectively increments by about 43.1%, 34.9%, and 106.5% for PMMA+Cu, PTFE+steel, and Al+Pb radioprotection disks at 10 MeV electron energy). Besides, the mean energy of the contaminant photons in the presence of each radioprotection disk is higher than the corresponding value in the disk absence. Both of these issues can be justified by increasing the severity of beam hardening through crossing the different layers of each considered shielding disk. The mean energy of the emerging photons from the lower surface of the Al+Pb disk at different intraoperative electron energies is higher than that of two other shielding disks which can be attributed to the higher photon attenuation and beam hardening effect induced by consecutive layers of this IOERT shielding disk. Furthermore, as expected, the mean energy of the contaminant photons increments with an increase in electron energy.

**FIGURE 5:**
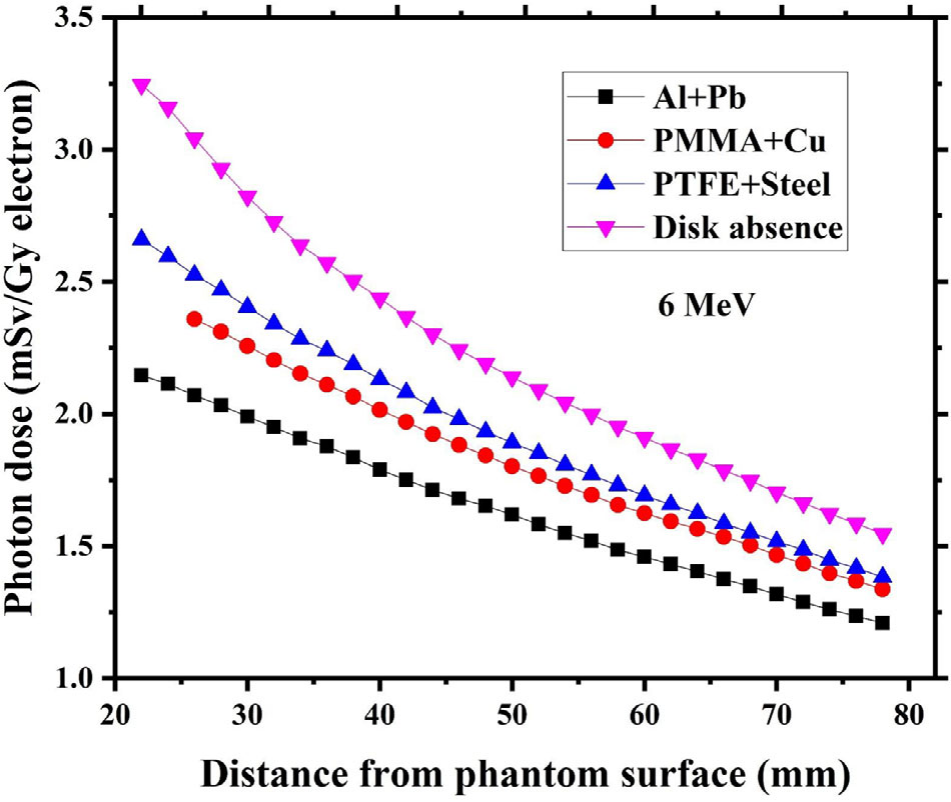
Contaminant photon dose values at different depths beneath each considered radioprotection disk at 6 MeV intraoperative electron energy. The corresponding calculated photon dose values at the absence of the disk at the same electron energy have been also depicted in this Figure. The reported dose values are relevant to the 1 Gy electron dose delivery at the depth of maximum dose inside the water.

**FIGURE 6:**
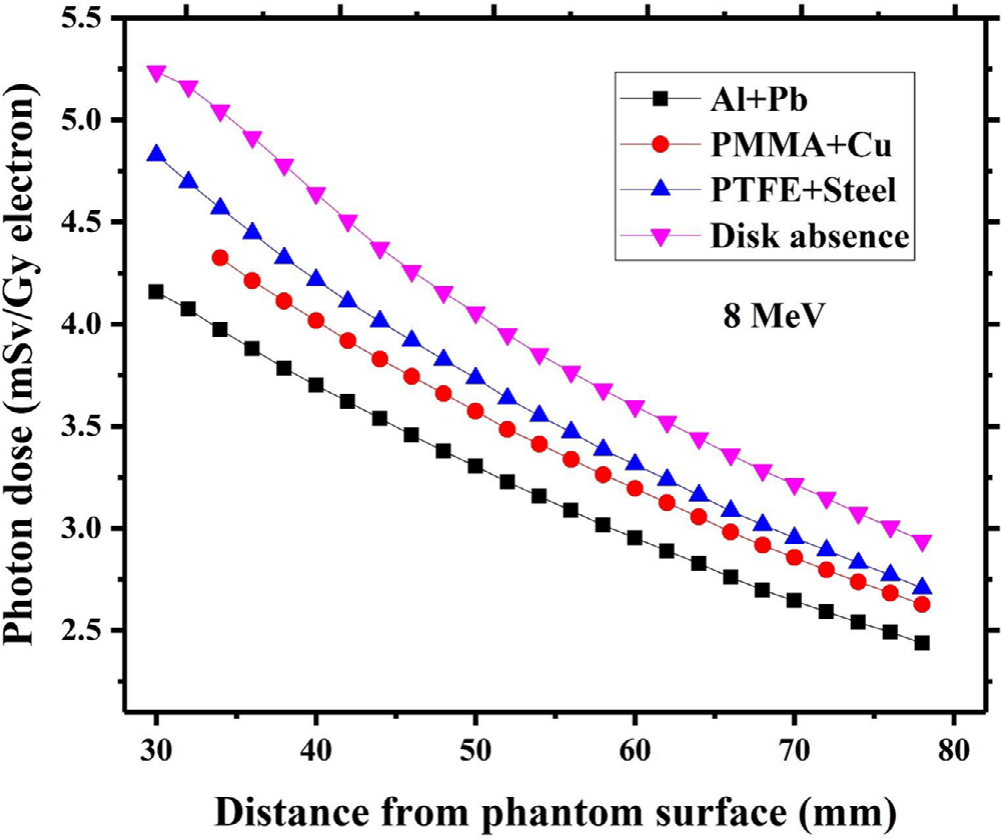
Contaminant photon dose values at different depths beneath each considered radioprotection disk at 8 MeV intraoperative electron energy. The corresponding calculated photon dose values at the absence of the disk at the same electron energy have been also depicted in this Figure. The reported dose values are relevant to the 1 Gy electron dose delivery at the depth of maximum dose inside the water.

**FIGURE 7:**
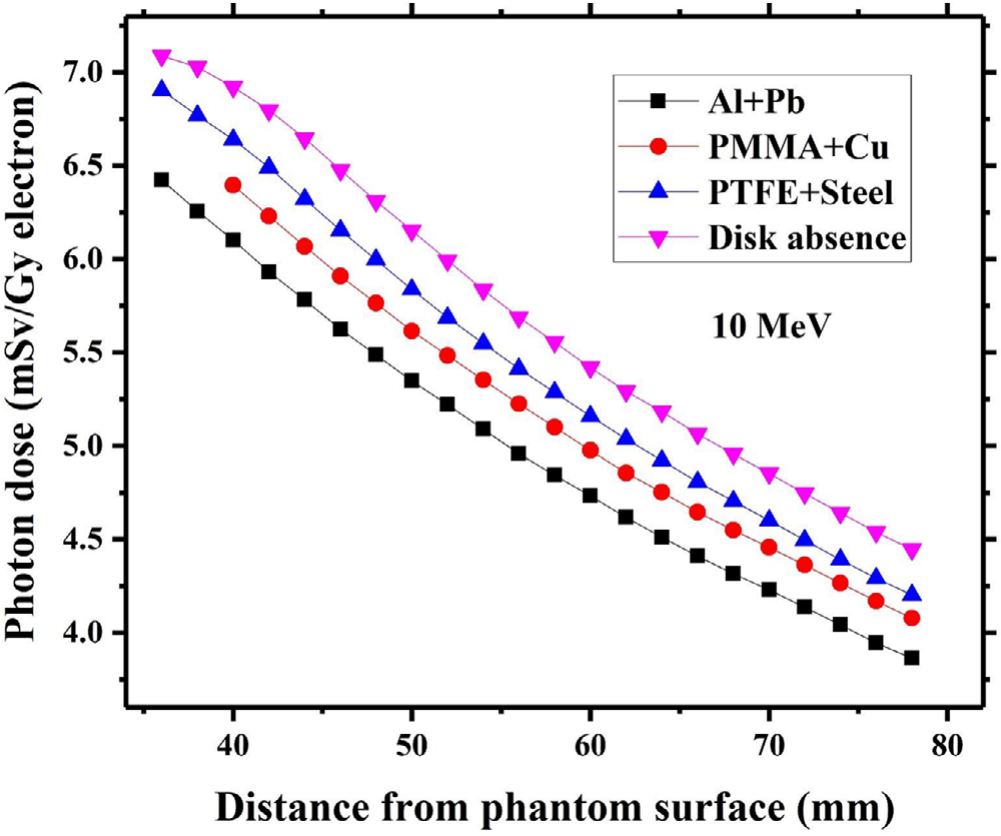
Contaminant photon dose values at different depths beneath each considered radioprotection disk at 10 MeV intraoperative electron energy. The corresponding calculated photon dose values at the absence of the disk at the same electron energy have been also depicted in this Figure. The reported dose values are relevant to the 1 Gy electron dose delivery at the depth of maximum dose inside the water.

**FIGURE 8:**
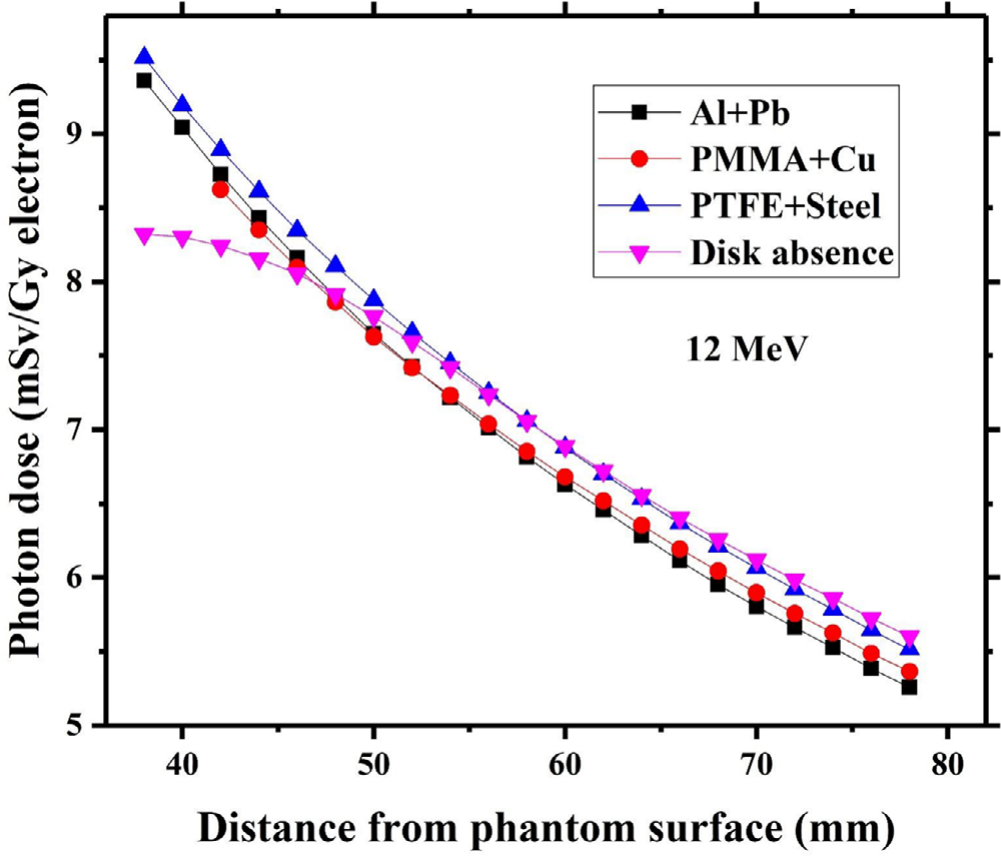
Contaminant photon dose values at different depths beneath each considered radioprotection disk at 12 MeV intraoperative electron energy. The corresponding calculated photon dose values at the absence of the disk at the same electron energy have been also depicted in this Figure. The reported dose values are relevant to the 1 Gy electron dose delivery at the depth of maximum dose inside the water.

Variations of induced dose by contaminant photons as a function of the depth beneath each studied radioprotection disk have been respectively drawn in Figures 5, 6, 7, 8 for intraoperative electron energies of 6, 8, 10, and 12 MeV. It should be noted that the reported dose values are corresponding to the administration of 1 Gy intraoperative electron dose at the depth of maximum dose (d_max_). This tuning was performed through dividing the calculated photon dose value at a given incident electron energy with the maximum electron dose along the central axis at the same energy.

As shown in Figure 5, the calculated contaminant photon dose values at 6 MeV electron energy in presence of each considered shielding disk are always lower than the estimated values in the disk absence. This trend is also found for 8 and 10 MeV electron energy, as illustrated in Figures 6 and 7. Such finding can be well justified by referring to the reported data in Figures 2, 3, 4. As depicted in Figures 2, 3, 4, the intensity of the contaminant photons just below each radioprotection disk is always less than the case where the disk is absent (this difference is more prominent at electron energies of 6–10 MeV). Therefore, it can be expected that the induced dose by contaminant photons reduces in presence of the IOERT shielding disk.

Comparing the obtained results in Figures 5, 6, 7 also demonstrates that the contaminant photon dose increments with an increase in the intraoperative electron energy. This fact is due to the increment of photon contamination intensity at higher electron energies (as shown in Figures 2, 3, 4). The minimum and maximum photon dose values at 10 MeV electron energy correspond to Al+Pb and PTFE+steel disk (6.4 mSv vs. 6.9 mSv). This finding can be also attributed to the fact that the intensity of contaminant photons reaches its minimum level beneath the Al+Pb disk, while the maximum intensities are found for the PTFE+steel disk.

Based on the presented results in Figures 5–7, it can be deduced that the calculated contaminant photon dose values in presence of each shielding disk get closer to the corresponding dose values at the disk absence when the intraoperative electron energy increases from 6 MeV to 10 MeV. This finding may be linked to the increment of contaminant photon intensity at higher electron energies and nearing their values to those which exist in the absence of the shielding disk, as illustrated in Figures 2, 3, 4.

On the other hand, as illustrated in Figure 8, the photon dose values in presence of the shielding disk can exceed the calculated ones in the disk absence at 12 MeV intraoperative electron energy (especially at close depths to the lower disk surface). Although the intensity of the contaminant photons at this electron energy is lower than that of disk absence, this intensity difference is now less than those observed at lower electron energies (for more clarification in this regard, refer to the reported data in Figures 2, 3, 4). In addition, as listed in Tables 2, 3, 4, the mean energy of the contaminant photons at the lower surface of the studied shielding disks dramatically increases at 12 MeV electron energy. Finally, these two factors cause the contaminant photon dose in the presence of the disk exceeds those obtained in the disk absence.

For 12 MeV electron energy, PMMA+Cu and PTFE+steel shielding disks respectively show the minimum and maximum contaminant dose values which can be also attributed to the intensity of the photon contamination beneath these two radioprotection disks.

According to the presented data in Figure 8, it can be realized that the maximum contaminant photon dose for PMMA+Cu, PTFE+steel, and Al+Pb is respectively equal to 8.6 mSv, 9.5 mSv, and 9.4 mSv for 1 Gy intraoperative electron dose delivery at d_max_. Assuming a 21 Gy dose prescription during breast cancer IOERT (following the radical breast IOERT treatment) at 90% isodose level,^18^, ^33^, ^34^, ^35^ the maximum received dose to the patient would be about 23.3 Gy. Consequently, it can be expected that the maximum contaminant photon dose which would be administered to the underlying healthy tissues in using the studied IOERT shielding is respectively equal to about 200, 221, and 219 mSv which are negligible amounts in comparison with the tolerance dose level of the healthy tissues.

Apart from photon contamination, the other concern about implementation of these shielding disks during breast intraoperative radiotherapy would be neutron production (or neutron contamination, in other words). The main origin of this neutron contamination is the photonuclear reaction of the contaminant photons with constructive elements of considered radioprotection disks. Nevertheless, it should be noted that photodisintegration is a threshold reaction and the photon energy must reach this threshold level for neutron production. Contributing elements to the PTFE+steel disk are Carbon (C), Fluorine (F), Silicon (Si), Chromium (Cr), Manganese (Mn), Iron (Fe), and Nickel (Ni). The constructive elements of the PMMA+Cu shield are also Hydrogen (H), Carbon (C), and Oxygen (O). The composing elements of the Al+Pb disk are also obvious. The minimum required photon energies for neutron production through interaction with the above‐mentioned elements have been listed by the IAEA‐TECDOC‐1178 report^36^ and relevant extracted values for these elements have been reported in Table 5.

**TABLE 5:** Minimum required photon energy for neutron production during the photonuclear reaction with Hydrogen (H), Carbon (C), Oxygen (O), Fluorine (F), Aluminum (Al), Silicon (Si), Chromium (Cr), Manganese (Mn), Iron (Fe), Nickel (Ni), and Lead (Pb) according to the released data by IAEA‐TECDOC‐1178 report [35].

Element											
	^2^H	^13^C	^15^O	F	^27^Al	^29^Si	^54^Cr	^55^Mn	^57^Fe	^61^Ni	207 Pb
**Natural abundance (%)**	0.01	1.11	0.04	N.A.	100	4.67	2.37	100	2.10	1.14	22.10
**Threshold photon energy (MeV)**	2.22	4.95	4.14	N.A.	13.06	8.47	7.72	10.23	7.65	7.82	6.74

It should be mentioned that the reported threshold energy levels in Table 5 are related to the minimum observed values for all naturally available isotopes of the survived elements to take into account the most possible case for neutron production. The corresponding isotope number and its natural abundance for which the minimum threshold energy is observed, have been also listed in Table 5 for each element.

Comparing the required photon energy level for neutron production (as listed in Table 5) in different constituents of studied radioprotection disks with the reported mean contaminant X‐ray energy values in Tables 2, 3, 4 reveals that the energy of the produced photons is much lower than the required threshold level for neutron production within the radioprotection disk body. Besides, the natural abundance of the candidate isotopes of the survived elements for neutron production is often low. Therefore, it can be expected that no neutron contamination would be associated with these IOERT shielding disks during breast cancer treatment. The results of our Monte Carlo‐based simulations also confirmed the accuracy of this hypothesis, so that no neutron trace was found during the performed simulations in the triple particle tracking mode of neutron‐photon‐ electron (n‐p‐e).

## 4 CONCLUSION

The induced photon contamination by some employed radioprotection disks during breast intraoperative electron radiotherapy including PMMA+Cu, PTFE+steel, and Al+Pb double‐layered ones were evaluated and compared in the current study following a Monte Carlo simulation approach.

The results showed none of the considered IOERT shielding disks will induce significant radiation contamination at electron energies up to 10 MeV, so that the X‐ray dose beneath each considered disk was lower than the case that the radioprotection disk was absent. This fact indicates that the evaluated radioprotection disks have a desirable operation in the absorption of produced contaminant X‐rays both within the water phantom and the disk body itself. The Al+Pb disk configuration has the best performance in the reduction of contaminant photon dose up to 10 MeV intraoperative electron energy.

On the other hand, the induced contaminant photon dose in presence of all considered shielding disks exceeds the correspondingly calculated X‐ray dose values in the disk absence at 12 MeV intraoperative electron energy. The PMMA+Cu disk configuration showed the best shielding characteristics in the reduction of the contaminant X‐ray dose which is produced at 12 MeV electron energy.

From the obtained results, it can be finally concluded that all of the radioprotection disks under investigation would produce slight X‐ray contamination, so that the contaminant X‐ray dose can be neglected in comparison with the prescribed intraoperative electron dose and threshold dose level of exposing healthy organs. Furthermore, most of the produced contaminant photons would be also reabsorbed by the employed radioprotection disks.

## AUTHOR CONTRIBUTIONS

Hamidreza Baghani: Conceptualization, Methodology, Writing—Review & Editing, Supervision, Project administration, analysis and interpretation of data. Mostafa Robatjazi: Study concept and design, analysis and interpretation of data, drafting of the manuscript, critical revision of the manuscript for important intellectual content.

## CONFLICT OF INTEREST STATEMENT

We wish to confirm that there are neither conflicts of interest associated with this manuscript nor significant financial support for this work that could have influenced its outcome.

## References

[1] Veronesi U , Orecchia R , Luini A , et al. Full‐dose intra‐operative radiotherapy with electrons (ELIOT) during breast‐conserving surgery: experience with 1246 cases. Ecancermedicalscience. 2008;2:65. published online ahead of print 2008/01/01. 22275962 10.3332/eCMS.2008.65 PMC3234040

[2] Rocco N , Rispoli C , Iannone L , et al. Intraoperative radiation therapy with electrons in breast cancer conservative treatment: our experience. Int J Surg. 2014;12:S75‐S78. 24862660 10.1016/j.ijsu.2014.05.049

[3] Sedlmayer F , Reitsamer R , Wenz F , et al. Intraoperative radiotherapy (IORT) as boost in breast cancer. Radiat Oncol. 2017;12(1):23. published online ahead of print 2017/01/21. 28103903 10.1186/s13014-016-0749-9 PMC5244574

[4] Kaiser J , Reitsamer R , Kopp P , et al. Intraoperative electron radiotherapy (IOERT) in the treatment of primary breast cancer. Breast Care (Basel). 2018;13(3):162‐167. published online ahead of print 2018/08/03. 30069175 10.1159/000489637 PMC6062668

[5] Dutta SW , Showalter SL , Showalter TN , Libby B , Trifiletti DM . Intraoperative radiation therapy for breast cancer patients: current perspectives. Breast Cancer (Dove Med Press). 2017;9:257‐263. published online ahead of print 2017/05/02. 28458578 10.2147/BCTT.S112516 PMC5402914

[6] Akbari ME , Nafissi N , Mahdavi SR , et al. Pros and cons of intraoperative radiotherapy: comparison of two clinical trials in breast cancer management. Int J Cancer Manag. 2018:e68915.

[7] Baghani HR , Moradmand H , Aghamiri SMR . Breast intraoperative radiotherapy: a review of available modalities, dedicated machines and treatment procedure. J Radiother Pract. 2018;18(1):98‐106. published online ahead of print 08/31.

[8] Hensley FW . Present state and issues in IORT physics. Radiat Oncol. 2017;12(1):37. published online ahead of print 2017/02/15. 28193241 10.1186/s13014-016-0754-z PMC5307769

[9] Paunesku T , Woloschak GE . Future directions of intraoperative radiation therapy: a brief review. Front Oncol. 2017;7:300. 29312882 10.3389/fonc.2017.00300 PMC5732937

[10] Palta JR , Biggs PJ , Hazle JD , et al. Intraoperative electron beam radiation therapy: technique, dosimetry, and dose specification: report of task force 48 of the radiation therapy committee, American association of physicists in medicine. Int J Radiat Oncol Biol Phys. 1995;33(3):725‐746. published online ahead of print 1995/10/15. 7558965 10.1016/0360-3016(95)00280-C

[11] Beddar AS , Biggs PJ , Chang S , et al. Intraoperative radiation therapy using mobile electron linear accelerators: report of AAPM radiation therapy committee task group no. 72. Med Phys. 2006;33(5):1476‐1489. published online ahead of print 2006/06/07. 16752582 10.1118/1.2194447

[12] Baghani HR , Aghamiri SM , Mahdavi SR , Akbari ME , Mirzaei HR . Comparing the dosimetric characteristics of the electron beam from dedicated intraoperative and conventional radiotherapy accelerators. J Appl Clin Med Phys. 2015;16(2):5017. published online ahead of print 2015/06/24. 26103175 10.1120/jacmp.v16i2.5017 PMC5690101

[13] Biggs P , Willett CG , Rutten H , Ciocca M , Gunderson LL , Calvo FA . Intraoperative electron beam irradiation: physics and Techniques. In: Gunderson LL , Willett CG , Calvo FA , Harrison LB , eds. Intraoperative Irradiation: Techniques and Results. Humana Press; 2011:51‐72. doi:10.1007/978-1-61779-015-7_3

[14] Robatjazi M , Baghani HR , Mahdavic SR , Felici G . Evaluation of dosimetric properties of shielding disk used in intraoperative electron radiotherapy: a Monte Carlo study. Appl Radiat Isot. 2018;139:107‐113. published online ahead of print 2018/05/12. 29751323 10.1016/j.apradiso.2018.04.037

[15] Baghani HR , Robatjazi M , Mahdavi SR . Comparing the performance of some dedicated radioprotection disks in breast intraoperative electron radiotherapy: a Monte Carlo study. Radiat Environ Biophys. 2020;59(2):265‐281. published online ahead of print 2020/04/08. 32253497 10.1007/s00411-020-00836-z

[16] Russo G , Casarino C , Arnetta G , et al. Dose distribution changes with shielding disc misalignments and wrong orientations in breast IOERT: a Monte Carlo ‐ GEANT4 and experimental study. J Appl Clin Med Phys. 2012;13(5):3817. published online ahead of print 2012/09/08. 22955646 10.1120/jacmp.v13i5.3817 PMC5718242

[17] Severgnini M , de Denaro M , Bortul M , Vidali C , Beorchia A . In vivo dosimetry and shielding disk alignment verification by EBT3 GAFCHROMIC film in breast IOERT treatment. J Appl Clin Med Phys. 2014;16(1):5065. published online ahead of print 2015/02/14. 25679150 10.1120/jacmp.v16i1.5065 PMC5689990

[18] Ciocca M , Orecchia R , Garibaldi C , et al. In vivo dosimetry using radiochromic films during intraoperative electron beam radiation therapy in early‐stage breast cancer. Radiother Oncol. 2003;69(3):285‐289. published online ahead of print 2003/12/04. 14644488 10.1016/j.radonc.2003.09.001

[19] Baghani HR , Robatjazi M , Mahdavi SR , Nafissi N , Akbari ME . Breast intraoperative electron radiotherapy: image‐based setup verification and in‐vivo dosimetry. Phys Med. 2019;60:37‐43. published online ahead of print 2019/04/20. 31000084 10.1016/j.ejmp.2019.03.017

[20] Oshima T , Aoyama Y , Shimozato T , et al. An experimental attenuation plate to improve the dose distribution in intraoperative electron beam radiotherapy for breast cancer. Phys Med Biol. 2009;54(11):3491‐3500. published online ahead of print 2009/05/14. 19436105 10.1088/0031-9155/54/11/014

[21] Heidarloo N , Baghani HR , Aghamiri SM , Mahdavi SR , Akbari ME . Commissioning of beam shaper applicator for conformal intraoperative electron radiotherapy. Appl Radiat Isot. 2017;123:69‐81. published online ahead of print 2017/03/07. 28260609 10.1016/j.apradiso.2017.02.039

[22] Robatjazi M , Tanha K , Mahdavi SR , et al. Monte Carlo simulation of electron beams produced by LIAC intraoperative radiation therapy accelerator. J Biomed Phys Eng. 2018;8(1):43‐52. published online ahead of print 2018/05/08. 29732339 PMC5928310

[23] Baghani HR , Robatjazi M , Mahdavi SR . Performance evaluation and secondary monitor unit checkout for a dedicated accelerator in intraoperative electron radiotherapy. Radiat Phys Chem. 2019;163:11‐17.

[24] Baghani HR , Robatjazi M , Mahdavi SR , Hosseini Aghdam SR . Evaluating the performance characteristics of some ion chamber dosimeters in high dose per pulse intraoperative electron beam radiation therapy. Phys Med. 2019;58:81‐89. published online ahead of print 2019/03/03. 30824155 10.1016/j.ejmp.2019.01.019

[25] Pelowitz D . MCNPXTM User's Manual. 2008.

[26] Osei‐Mensah W , Fletcher JJ , Danso KA . MCNPX simulated bremsstrahlung X‐ray spectrum at 5 MeV, 10 MeV and 15 MeV electron beam interaction with tungsten target. Int Res J Eng Technol. 2019;06(08):1114‐1118.

[27] Baghani HR , Hosseini Aghdam SR , Robatjazi M , Mahdavi SR . Monte Carlo‐based determination of radiation leakage dose around a dedicated IOERT accelerator. Radiat Environ Biophys. 2019;58(2):263‐276. 30972494 10.1007/s00411-019-00786-1

[28] Alhamada H , Simon S , Philippson C , et al. Shielding disk position in intra‐operative electron radiotherapy (IOERT): a Monte Carlo study. Phys Med. 2018;51:1‐6. published online ahead of print 2018/10/04. 30278980 10.1016/j.ejmp.2018.05.023

[29] Alhamada H , Simon S , Philippson C , et al. 3D Monte Carlo dosimetry of intraoperative electron radiation therapy (IOERT). Phys Med. 2019;57:207‐214. published online ahead of print 2019/02/11. 30738527 10.1016/j.ejmp.2018.12.037

[30] Alhamada H , Simon S , Philippson C , et al. Monte Carlo dose calculations of shielding disks with different material combinations in intraoperative electron radiation therapy (IOERT). Cancer Radiother. 2020;24(2):128‐134. published online ahead of print 2020/04/01. 32224107 10.1016/j.canrad.2020.02.006

[31] Baghani HR , Aminafshar B . In‐field radiation contamination during intraoperative electron radiation therapy with a dedicated accelerator. Appl Radiat Isot. 2020;155:108918. published online ahead of print 2019/10/08. 31590100 10.1016/j.apradiso.2019.108918

[32] Protection R . ICRP publication 103. Ann ICRP. 2007;37(2.4):2. 10.1016/j.icrp.2007.10.003 18082557

[33] Forouzannia A , Harness JK , Carpenter MM , et al. Intraoperative electron radiotherapy boost as a component of adjuvant radiation for breast cancer in the community setting. Am Surg. 2012;78(10):1071‐1074. published online ahead of print 2012/10/03. 23025943

[34] Fastner G , Sedlmayer F , Merz F , et al. IORT with electrons as boost strategy during breast conserving therapy in limited stage breast cancer: long term results of an ISIORT pooled analysis. Radiother Oncol. 2013;108(2):279‐286. published online ahead of print 2013/07/09. 23830467 10.1016/j.radonc.2013.05.031

[35] Leonardi M , Cecconi A , Luraschi R , et al. Electron beam intraoperative radiotherapy (ELIOT) in pregnant women with breast cancer: from in vivo dosimetry to clinical practice. Breast Care (Basel). 2017;12(6):396‐400. published online ahead of print 2018/02/20. 29456472 10.1159/000479862 PMC5803713

[36] Chadwick M , Oblozinsky P , Blokhin A , et al. Handbook on photonuclear data for applications: cross sections and spectra. IAEA Tech‐Doc. 2000:1178.

